# Structural and Functional Characterization of Ribosomal Protein Gene Introns in Sponges

**DOI:** 10.1371/journal.pone.0042523

**Published:** 2012-08-06

**Authors:** Drago Perina, Marina Korolija, Andreja Mikoč, Maša Roller, Bruna Pleše, Mirna Imešek, Christine Morrow, Renato Batel, Helena Ćetković

**Affiliations:** 1 Department of Molecular Biology, Rudjer Boskovic Institute, Zagreb, Croatia; 2 Department of Molecular Medicine, Rudjer Boskovic Institute, Zagreb, Croatia; 3 Department of Molecular Biology, Faculty of Science University of Zagreb, Zagreb, Croatia; 4 School of Biological Sciences, Queen's University, Belfast, United Kingdom; 5 Center for Marine Research, Rudjer Boskovic Institute, Rovinj, Croatia; University of Münster, Germany

## Abstract

Ribosomal protein genes (RPGs) are a powerful tool for studying intron evolution. They exist in all three domains of life and are much conserved. Accumulating genomic data suggest that RPG introns in many organisms abound with non-protein-coding-RNAs (ncRNAs). These ancient ncRNAs are small nucleolar RNAs (snoRNAs) essential for ribosome assembly. They are also mobile genetic elements and therefore probably important in diversification and enrichment of transcriptomes through various mechanisms such as intron/exon gain/loss. snoRNAs in basal metazoans are poorly characterized. We examined 449 RPG introns, in total, from four demosponges: *Amphimedon queenslandica*, *Suberites domuncula*, *Suberites ficus* and *Suberites pagurorum* and showed that RPG introns from *A. queenslandica* share position conservancy and some structural similarity with “higher” metazoans. Moreover, our study indicates that mobile element insertions play an important role in the evolution of their size. In four sponges 51 snoRNAs were identified. The analysis showed discrepancies between the snoRNA pools of orthologous RPG introns between *S. domuncula* and *A. queenslandica*. Furthermore, these two sponges show as much conservancy of RPG intron positions between each other as between themselves and human. Sponges from the *Suberites* genus show consistency in RPG intron position conservation. However, significant differences in some of the orthologous RPG introns of closely related sponges were observed. This indicates that RPG introns are dynamic even on these shorter evolutionary time scales.

## Introduction

The ribosome is a protein-RNA complex, fundamentally the same in all three domains of life, and is the crucial cell component for protein synthesis. X-ray crystal structures of ribosomal subunits were used to determine ribosome structure and function in detail [1]–[3]. Ribosome assembly is a complex process that includes coordinated activation of more than 200 non-ribosomal factors and many small nucleolar RNAs (snoRNAs), modification of ribosomal RNA (rRNA) and its correct assembly with ribosomal proteins (RPs) [4], [5]. RPs are evolutionarily conserved [6], [7]. 50 to 54 RPs have been found in eubacteria, 57 to 68 in archaea and 79 to 81 in eukaryotes [8], while the analysis of 66 complete genomes revealed that 34 RPs are common to all living organisms [7]. Many RPs possess additional, extraribosomal functions in cells [9]. They are involved in many processes within the ribosome system, surveillance of ribosome synthesis but also in replication and regulation of cell growth, apoptosis and cancer [8]. Due to the presence of practically the same RPs in all eukaryotes and their ancient origin, ribosomal protein genes (RPGs) are a suitable model for studying intron dynamics [10]. A recent study on RPGs showed that trends of intron gain and loss differ across species in a given kingdom, but appear to be more consistent within subphyla [11]. Analysis of a partial set of highly conserved intron sites in the genome of the sponge *Amphimedon queenslandica* revealed that intron position and phase are conserved relative to other metazoans [12]. The role of introns in eukaryotic genomes is still not well understood. Some non-protein-coding RNAs (ncRNAs) are transcribed from introns in protein-coding or non-protein-coding genes. The accumulating genomic data strongly confirm tendency of snoRNAs to colonize RPGs and ribosome related genes in eukaryotes [13] and suggest that these genes have conserved snoRNAs across mammals [14]. These snoRNAs are involved in the modification of rRNAs, small nuclear RNAs (snRNAs) and transfer RNAs (tRNAs) in archaea [15]–[17]. Recent findings have demonstrated that snoRNAs can also target mRNAs and may possess microRNA-like functions [18]–[20]. The major classes of snoRNAs include C/D box snoRNAs, which primarily guide the 2′-O-methylation of target rRNAs; H/ACA box snoRNAs, which typically guide pseudouridylation of target rRNAs and small Cajal-body-specific RNAs (scaRNAs) which typically target snoRNAs. snoRNAs in basal metazoans are still poorly characterized. Because snoRNAs were identified in Archaea and Eukarya, it was surprising that the only systematic ncRNA genome annotation among basal metazoans found merely eight snoRNAs, showing that host genes of snoRNAs in *Trichoplax adhaerens* are not conserved in human [21]. This number is exceedingly lower than in “higher” animals. This is in accordance with merely eight microRNAs (miRNAs) identified in the sponge *A. queenslandica*, which may indicate that metazoan complexity correlates with an increasing number of miRNAs [22]. The observation that recent introns, which are present in the human *Nme6* gene, but not in the sponge ortholog, contain miRNAs also supports that thesis [23]. snoRNAs are believed to be the most ancient ncRNAs [24]. Many examples of ncRNAs displaying both snoRNA and miRNA characteristics suggest a possible evolution from one type to the other [25]. snoRNAs are mobile genetic elements, often transferred through retrotransposition, and can therefore participate in diversification and enrichment of transcriptomes through various mechanisms such as intron/exon gain/loss [26]. Sponges (Porifera) are basal metazoans which branched off first from the common ancestor of all animals. Recent analysis showed that sponges are similar to other animals in terms of genome content, structure and organization and that sponges have a wide repertoire of genes, many of which are involved in diseases in more complex metazoans [12], [27]. Good examples are biochemical properties and biological functions of a vertebrates' non-metastatic multifunctional enzyme present in sponges, which suggest evolutionary origin of these traits even before the appearance of true tissues and the origin of tumors and metastasis [28]. RPs do not appear to be an exception. The overall sequence conservation between sponge and rat RPs is 80% or higher [29]. Furthermore, *cis*-regulatory architecture in promoters of RPGs appears not to be drastically changed from sponge to human [30]. Available data suggest that sponges changed little during metazoan evolution and are probably the most plausible model for studying gene/protein structure in ancestral metazoan. Our goal was to characterize intron dynamics in sponge RPGs by checking the intron sequences for the presence of snoRNAs and other over-represented elements. Because many snoRNAs and miRNAs have been found to exist in one or a small number of organisms, suggesting that they are dynamic and have a fast evolving nature, they are ideal for providing insight into dynamics of sponges' RPG introns, and show how they changed during metazoan evolution. Our results show that sponge *A. queenslandica* RPG introns are similar in many structural characteristic to “higher” metazoans. Furthermore, the sponges *A. queenslandica* and *S. domunucula* show as much conservancy of RPG intron positions between each other as between themselves and human. However, when we compared RPG introns with three species from the genus *Suberites* of sponges, there was a discrepancy in snoRNA pools between the genus *Suberites* and *A. queenslandica*. Even though the intron positions in the *Suberites* genus are conserved, there are significant differences in certain RPG introns which indicate that they are dynamic even on these shorter evolutionary time scales.

## Materials and Methods

To identify *A. queenslandica* expressed sequence tags (ESTs) encoding homologs of human RPs TBLASTN (NCBI, NIH, Bethesda, MD, USA: http://www.ncbi.nlm.nih.gov) was used. The set of sponge RPGs was obtained using the NCBI WGS (whole genome shotgun) Trace Archive as well as the assembled draft genome available at http://spongezome.metazome.net/cgi-bin/gbrowse/amphimedon/ [12]. The GC content of RPGs was calculated by CODONW (http://codonw.sourceforge.net/), while the GC content of the whole genome was calculated using geecee from the EMBOSS suite [31]. ClustalX [32] was used for multiple alignments of RPs from sponge and their orthologs as well as for alignments of concatenated RPs from sponge and nine other species (*Homo sapiens, Strongylocentrotus purpuratus, Drosophila melanogaster, Caenorhabditis elegans, Nematostella vectensis, Trichoplax adhaerens, Monosiga brevicollis, Saccharomyces cerevisiae* and *Arabidopsis thaliana*). One RPG from *T. adhaerens*, four from *N. vectensis* and ten from *M. brevicolis* were assembled manually from data available on the NCBI Trace Archive to give a total set of 55 orthologs from nine organisms. Due to the lack of a fully annotated set of RPGs from the nine organisms mentioned, it was not possible to analyze the entire set. RPGs analyzed are listed in Table S1. Statistical data were extracted from GeneDoc (http://www.psc.edu/biomed/genedoc). MEME was used for searching for over-represented motifs in RPG introns [33]. MEME searches for the most significant motifs in the input sequences and reports an E-value for each motif it finds. For this study, the search was limited to finding the top 30 motifs that are 5–50 bp long. To identify snoRNAs in introns of RPGs snoSeeker was used [34]. To check whether snoRNAs match known snoRNAs' motifs we used Rfam [35], the snOPY database (http://snoopy.med.miyazaki-u.ac.jp/) and the snoRNA-LBME database [36]. The secondary structure of snoRNAs was computed using the program RNAfold from the Vienna RNA Package [37]. To check for presence of snoRNAs in sponge *Suberites domuncula* RPGs, the corresponding and neighboring introns were sequenced. All primer sequences used in this study are given in Table S2. The same primers were used to sequence the corresponding RPG introns of *Suberites ficus* and *Suberites pagurorum* from genomic DNA isolated as previously described [38]. Both the concatenated sequences from each organism and individual introns were aligned using ClustalX [32] and statistics on the alignments extracted through the infoalign program of the EMBOSS suite [31]. For isolation of small RNAs, fresh specimens of *S. domuncula* were cut into pieces, frozen in liquid nitrogen and ground to a fine powder. Approximately 3 µg of small RNAs were obtained from 260 mg of tissue powder by the mirPremier microRNA Isolation Kit (Sigma), according to the manufacturer's protocol for plant tissue. Polyadenilation of 1 µg of small RNAs was achieved by incubation with 5 U of the *E. coli* Poly(A) Polymerase (BioLabs) for 15 minutes at 37°C. All 12 µl of the poly(A) tailing reaction mixture was then reverse transcribed using the SuperScript II Reverse Transcriptase (Invitrogen) and a modified poly-d(T) primer (modpolydT). The resulting cDNA was diluted to 100 µl and each PCR was performed on 3 µl of cDNA using the HotStarTaq DNA Polymerase (Qiagen), a universal reverse primer (uni) and forward primers specific for predicted snoRNAs. The products were cloned into the pGEM-T vector (Promega). Positive clones were sequenced using the ABI PRISM BigDye Terminator v3.1 Ready Reaction Cycle Sequencing Kit and T7/pUC primers.

## Results and Discussion

### Structural characterization of sponge A. queenslandica RPG introns

**Figure 1:**
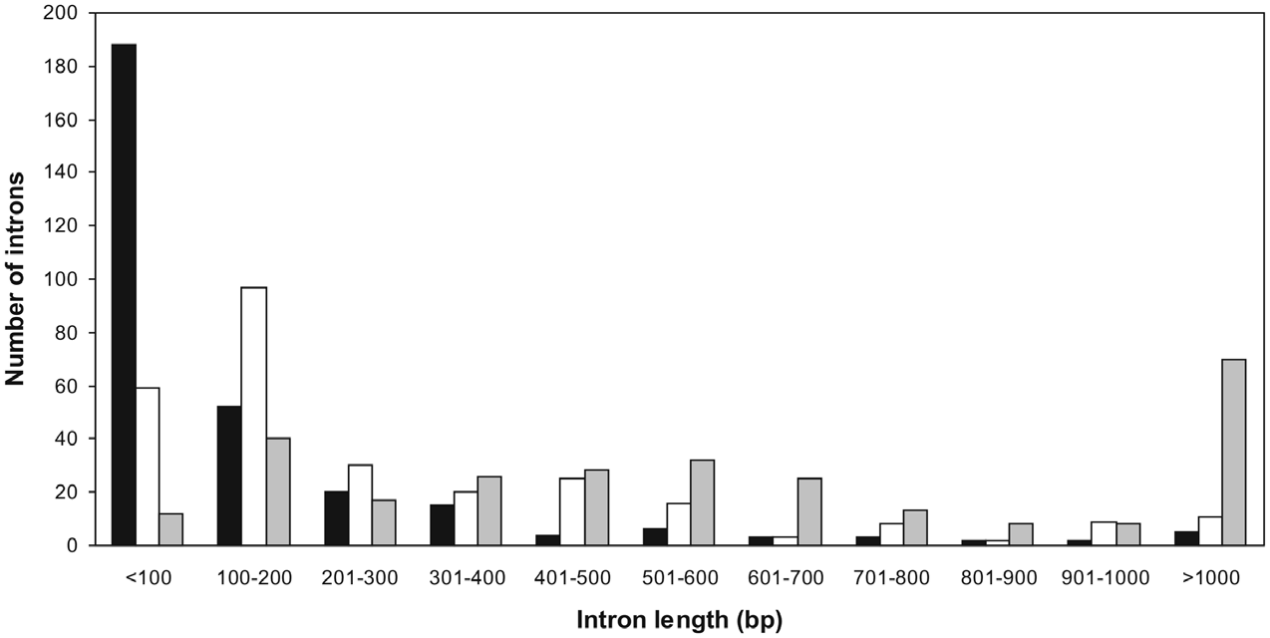
Size distribution of the RPG introns in sponge *A. queenslandica* (black bars), cnidarian *N. vectensis* (gray bars), and placozoan *T. adhaerens* (white bars).

**Figure 2:**
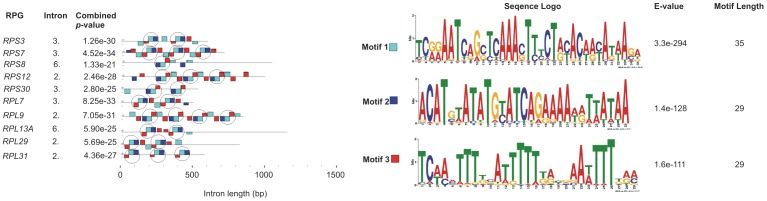
Over-represented elements in sponge *A. queenslandica* RPG introns composed of three motifs. Full tripartite motifs are circled. The E-value and combined p-value were extracted from MEME.

Full-length cDNA sequences coding for 79 RPs were identified in the marine sponge *A. queenslandica* (AQ) genome, and the gene structure for 78 of them was completely ascertained. The *RPS14* gene was not completely determined due to the absence of WGS sequences indispensable for assembling one long intron. This gene was not considered in calculating the average values shown in Table S3. 76 RPGs from cnidarian *N. vectensis* and 73 from placozoan *T. adhaerens* were used as control. Sponge AQ RPGs contained an average of 4.01 introns. The *RPP0* gene had the largest number of introns - 10, while the only gene without introns was *RPL35* (Table S3). In 78 complete RPGs a total of 312 introns with an average length of 164 bp were found. However, three quarters of the introns were shorter than the average value (Fig. 1), which indicates that only a few long ones contribute considerably in accretion of average intron length. The median value of RPG intron length was 68 bp, which is slightly lower than the median intron length for the published draft genome (81 bp) [12]. The longest was the second intron of the *RPS27* gene (2263 bp), and the shortest one was the first intron of the *RPS21* gene (37 bp). Human RPGs have significantly longer introns of 760 bp on average [39]. Transposable element insertions play an important role in the evolution of intron size [40]. Therefore we checked for over-represented elements in sponge AQ RPG introns. We found 24 copies of a tripartite element in ten introns of ten RPGs present in one to four copies (Fig. 2). The average intron length of these ten introns was 780 bp, and each one of them is longer than 500 bp. Only 23 of 312 sponge RPG introns are longer than 500 bp, which indicates that these element insertions contribute to sponge AQ RPG intron length. The average coding sequence (CDS) length did not differ as drastically as intron length. AQ had an average of 504 bp long CDSs, while human had 521 bp [39]. Most AQ introns, 285 of them, were found between translational start and stop codons, 26 introns were found in the 5′ untranslated region (5′ - UTR) and only one in the 3′ UTR of the *RPS9* gene. Most of the introns found between translational start and stop codons, were phase 0 (52%), 27% were phase 1 and 21% were phase 2. These results support the so-called “50/30/20 rule” of intron phase distributions. It has been found that across many studied organisms, approximately 50% of introns are phase 0, 30% are phase 1 and 20% are phase 2 [41]. Almost all introns found in sponge AQ RPGs start with GT and end with AG (so called GT-AG introns). Only 2% were GC-AG introns, and AT-AC introns were not found. The average guanine and cytosine (GC) content of introns was 31.2%, which is considerably smaller than the GC content of coding sequences (44.2%). Moreover, a higher GC content in exons was observed in every RPG, without exception (Table S3). A similar effect has been found in human, where exons generally also have higher GC content than introns and intergenic regions [42]. It has also been shown that the sponge *S. domunucula* (SD) RPGs have a preference for C- and G- ending codons [43] and that the genome has a GC content of 39%. Based on our estimate of a similar amount of GC content (36%) in the genome of AQ we predict that this effect is probably also pronounced in this Demospongiae.

**Figure 3:**
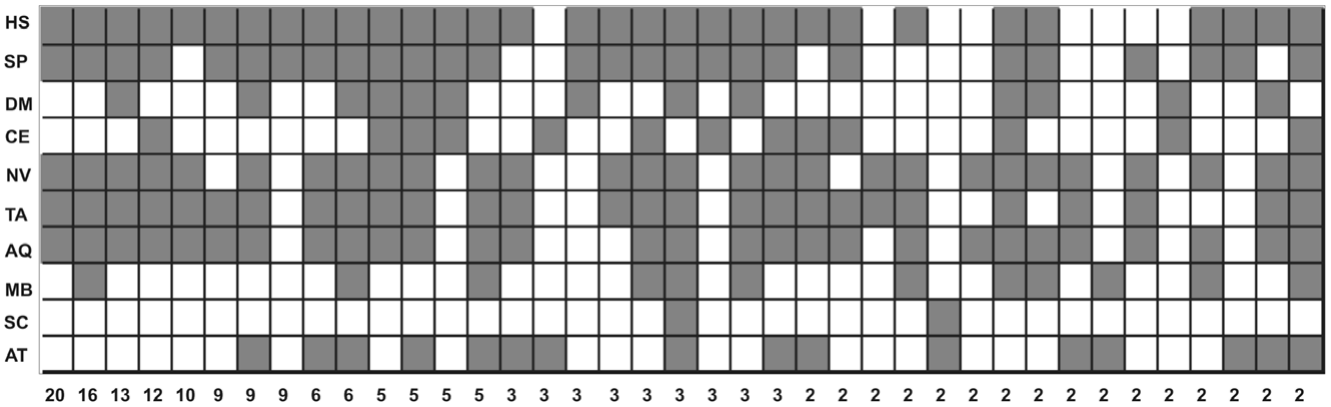
Combinatorial grouping of 10 species according to the number of shared introns . Gray squares indicate presence of introns.

To further explore introns' characteristics, 55 RPGs from sponge AQ were compared with orthologs from each of the following nine organisms whose whole genomes have been sequenced: *H. sapiens* (HS), *S. purpuratus* (SP), *D. melanogaster* (DM), *C. elegans* (CE), *N. vectensis* (NV), *T. adhaerens* (TA), *M. brevicollis* (MB), *S. cerevisiae* (SC) and *A. thaliana* (AT). The total number of analyzed introns within the coding regions of the RPGs in ten organisms was 1491 (Table S4). There are significant differences in intron number and length among these organisms causing variation in gene size. Characteristics of each RPG from model organisms used in this research (that are not included in Table S3) are given in Table S1. The position and phase of RPG introns were also compared in these ten organisms. The highest ratio of “unique” introns, those that are specific for a particular species, was found in yeast, 79.4%, and the lowest in placozoan, 3.1%. In all analyzed metazoans, except fruit fly and nematode worm, the ratio of “unique” introns was less than 10% (Table S4). Most positions, phases, and numbers of RPG introns, as well as RPs themselves (Table S5), were not drastically changed in metazoans from sponge to human. Fruit fly and nematode worm are the exceptions. 84.8% of sponge RPG introns are found in humans and 76.2% of human RPG introns are also present in sponge (Table S6). Our results support previously observed extensive intron loss in fruit fly and nematode worm [44]. Intron-sharing among all ten organisms is shown in Fig. 3. The highest number of RPG introns is shared between human, sea urchin, sea anemone, placozoan and sponge. The same organisms occur in other highly represented combinations of shared introns.

Sponge AQ RPG introns show similarity with other metazoans in exon/intron GC content, they follow the so-called “50/30/20 rule”, mobile element insertions play an important role in the evolution of their size and show position conservancy with other metazoans, which may indicate their functional importance.

### Identification of snoRNAs in RPG introns of sponges

**Figure 4:**
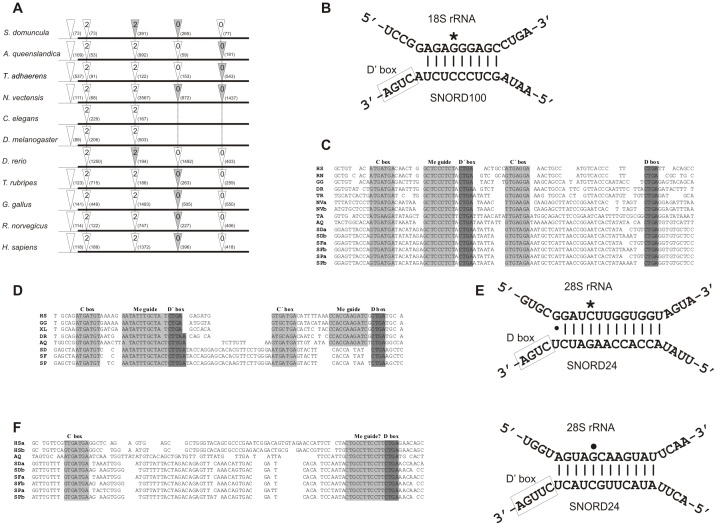
C/D box snoRNAs conserved in sponges and human. (A) Intron-mapping of *RPS12* genes from representative species. White triangles indicate positions of the introns and gray triangles indicate presence of SNORD100. The number in the triangle denotes the intron phase and the number in brackets intron length. The thin line indicates the 5′ UTR region. (B) The target site of SNORD100 in 18S rRNA is marked with an asterisk. (C) Alignment of SNORD100 orthologs from various metazoans. (D) Alignment of SNORD24 orthologs. One methylation guide site in *S. domuncula*, *S. ficus* and *S. pagurorum* is not conserved. (E) Target sites of SNORD24 are conserved in sponge and marked with dot and asterisk. (F) Orthologs of SNORD83 with conserved methylation (Me) guide site of unknown target.

**Figure 5:**
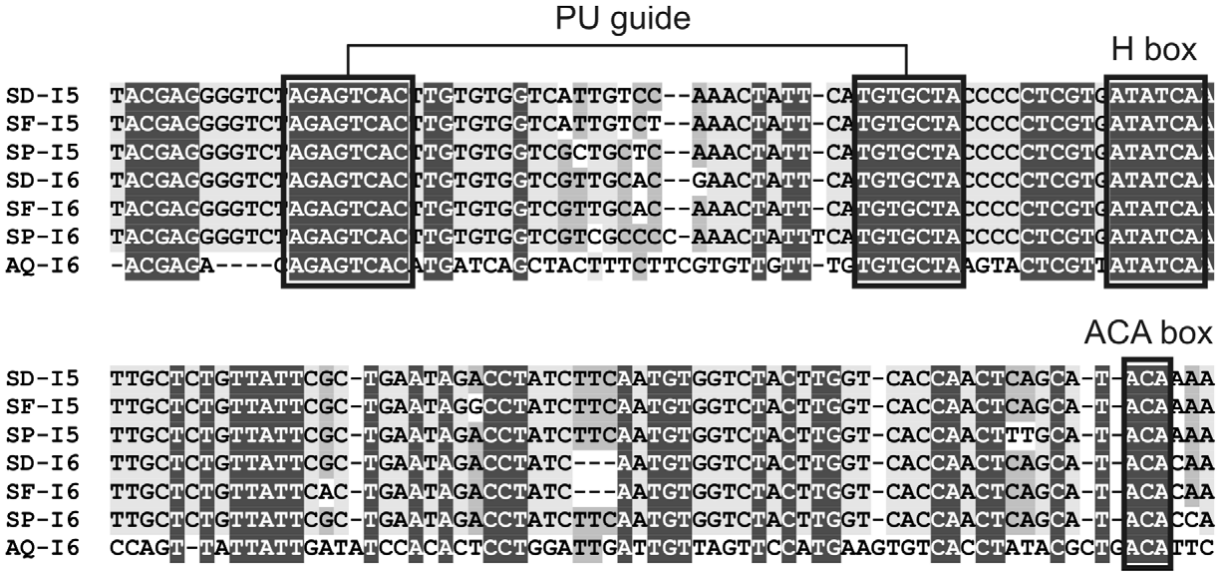
H/ACA snoRNAs conserved in the fifth and sixth introns (I5, I6) of the *RPL13A* gene in *S. domuncula* (SD), *S. ficus* (SF) and *S. pagurorum* (SP) and in the sixth intron of the same gene in *A. queenslandica* (AQ). All essential snoRNA elements are conserved and a putative pseudouridylation (PU) guide site is designated.

**Figure 6:**
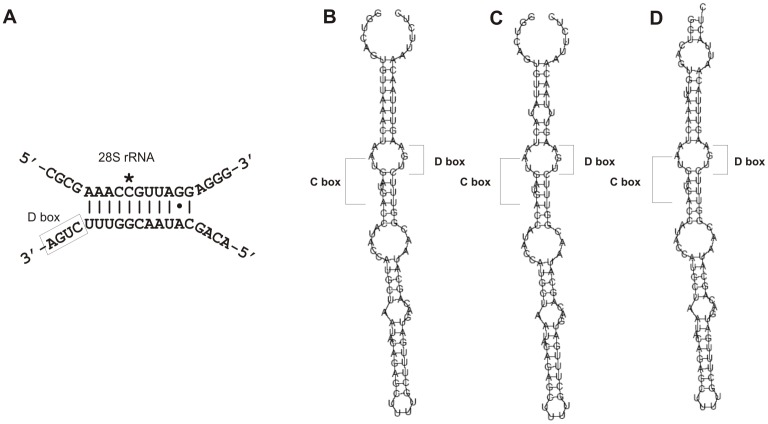
A potential 28S rRNA target (A) and secondary structure of a novel snoRNA found in the last intron of the *RPS19* gene in *S. domuncula* (B), *S. ficus* (C) and *S. pagurorum* (D).

Many snoRNAs were found within introns of vertebrate RPGs. In human, 57 snoRNAs were identified within introns of 28 RPGs [36]. The initial search in 316 introns of 79 sponge AQ RPGs, for which we used snoSeeker, with non-stringent search parameters, produced a candidate set of 16 C/D box snoRNAs and 2 H/ACA snoRNAs (Table S7). The corresponding 17 introns, as well as the neighboring ones, were sequenced in sponge SD to determinate dynamics of snoRNAs in sponges. The total set of 53 introns produced a candidate set of 9 C/D box snoRNAs and 2 H/ACA snoRNAs (Table S7). Furthermore, the corresponding 40 introns were sequenced in *S. ficus* (SF) and *S. pagurorum* (SP) which produced a candidate set of 9 C/D box snoRNAs and 2 H/ACA snoRNAs in each of the sponge (Table S7). With a more detailed analysis we were able to identified only three snoRNAs in all four sponges that match a sequence motif of known snoRNAs available on Rfam, the snOPY database and/or the snoRNA-LBME database. The first snoRNA is the sponge ortholog of the human C/D box snoRNA SNORD100 (HBII-429) found in the *RPS12* gene. We analyzed introns of *RPS12* genes in vertebrates (*Rattus norvegicus*, *Gallus gallus*, *Danio rerio, Takifugu rubripes*) and invertebrates (*D. melanogaster*, *C. elegans*, *N. vectensis*, *T. adhaerens*) and checked for the presence and the position of the SNORD100 ortholog. There is an obvious tendency of this snoRNA to colonize the *RPS12* gene from basal metazoans to “higher” vertebrates (Fig. 4A). Only in animals with intensive intron loss, SNORD100 was not identified in the *RPS12* gene. Its expression was verified experimentally. SNORD100 is predicted to guide the 2′-O-ribose methylation of guanine at position 436 in human 18S rRNA [45]. This target sequence of 18S rRNA is highly conserved between human and sponges investigated (Fig. 4B). The sponge SNORD100 methylation guide sequence as well as the C/D box were also well conserved (Fig. 4C). The other conserved snoRNA was found in the third intron of the *RPL5* gene in sponge AQ and in the same intron of SD, SF and SP *RPL5*. This snoRNA is an ortholog of the human C/D box snoRNA SNORD24 (U24), found in the second intron of the *RPL7A* gene. It is predicted to guide the 2′-O-ribose methylation of 28S rRNA cytosines at position 2338 and 2352 in human [46]. Sponge orthologs show different levels of conservation with human SNORD24 elements (Fig. 4D). Only one methylation guide site is conserved in all four sponges while target sequences of 28S rRNAs are well conserved in all of them. Interestingly, we confirm expression of this snoRNA in SD, which indicates a possible appearance of a novel snoRNA target or just loss of an old one. The third conserved snoRNA was found in the fourth intron of the sponge AQ *RPP0* gene and is most similar to human SNORD83A/SNORD83B (U83A/U83B) found in the fifth and seventh intron of the human *RPL3* gene, respectively (Fig. 4F). In SD, SF and SP this snoRNA was found in the second and last introns of the *RPP0* gene, but not in the intron that contains this snoRNA in AQ. More interestingly, another snoRNA is located in the last intron of the *RPP0* gene of AQ. Target RNA(s) of this snoRNA are still unknown [36]. All sponges have H/ACA box snoRNAs conserved in the *RPL13A* gene. While AQ possesses only one copy, in SD, SF and SP this snoRNA was found duplicated in the neighboring intron. The SD copies are 94% identical to each other, while one shares only 48% and the other 49% identical nucleotides with those from AQ. Although overall not well conserved, all essential snoRNA elements and target sites are maintained (Fig. 5). Some of the other snoRNAs, whose expression was verified experimentally show stable snoRNA secondary structures with conserved snoRNA parts. In the last intron of the *RPS19* gene the single snoRNA with a potential target rRNA was found (Fig. 6). This target has not yet been described as a methylation site in human so we can only speculate about the possible function of this snoRNA.

**Figure 7:**
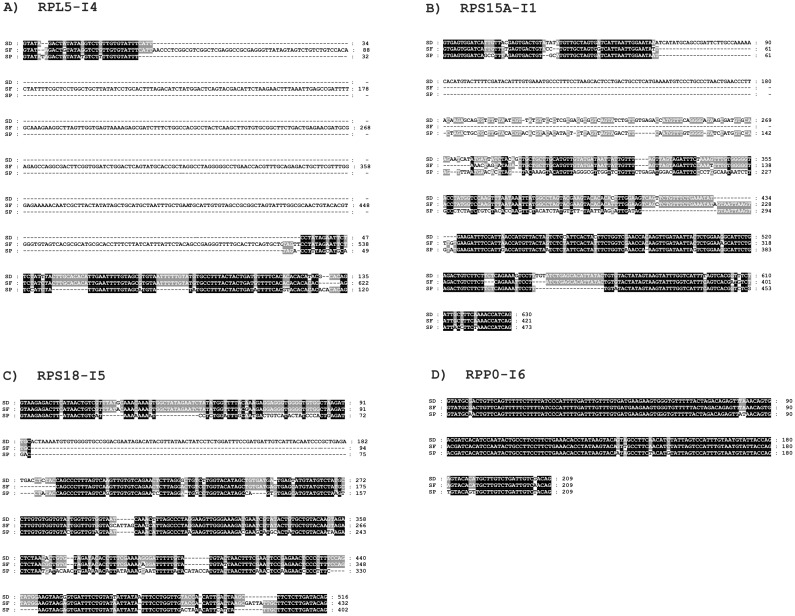
Multiple alignment of selected introns from three species of the genus *Suberites*: *S. domuncula* (SD), *S. ficus* (SF) and *S. pagurorum* (SP). A large insertion is shown in the fourth intron of the SF *RPL5* gene (A), changes in first introns of *RPS15A* gene (B), smaller changes between fifth introns of the *RPS18* gene (C) and relatively high conservation in the sixth intron of *RPP0* gene (D).

Both *trans*-duplication, duplication of snoRNA from introns to distant genomic locations, and previously mentioned *cis*-duplication, duplication of snoRNA to a neighboring intron of the same gene, were already observed in, most notably, nematodes [47] and platypus [48]. This is in accordance with a model for the evolutionary origin of guide snoRNAs which states that the major source of novel snoRNAs are duplications of the ancestral snoRNA gene [49]. It is interesting to note that all of the 18 identified snoRNAs in the sponge AQ were present in single copies in RPGs while in SD, SF and SP three were present in a single copy and four were found duplicated in neighboring introns. The diverse patterns of snoRNA loci in different sponges' RPGs are possibly a consequence of the mobility of snoRNAs [50]. One of the mechanisms has been shown to be retroposition [47], [48]. It is known that snoRNAs can change their genomic location even within relatively short (vertebrate) evolutionary time scales [51]. Mobile genetic sequences play an important role in the diversification of mammalian genomes, for example, through mechanisms such as exonization and intronization [40]. These mechanisms are probably also present in sponges. Differences in snoRNA pools in the orthologous RPGs of the SD and AQ sponges is also accompanied with differences in conservation of intron position. 87.5% of SD RPG introns analyzed were present in AQ, while 86.7% of SD RPG introns were present in human. These two Demospongiae belong to different clades, AQ to marine Haplosclerida (G3) and SD to the G4 clade [38]. Although it is difficult to resolve the exact time of the split of these clades, it has been estimated to have occurred 600 million years ago [52]. To examine dynamics of sponges' RPG introns on a smaller time scale, we sequenced the introns of two additional species from the *Suberites* genus: *S. ficus* (SF) and *S. pagurorum* (SP). The total of 126 RPG introns examined (available as Text S1) in these three more closely related species are 79.7%, 80.3% and 64.7% (for SD, SF an SP, respectively) conserved in relation to the consensus sequence from all three species (see Fig. S1). All examined RPG intron positions were conserved in *Suberites* species, which indicates that on this shorter evolutionary time scale mobility of snoRNAs is not a significant factor that determines intron dynamics. However, some introns possess significant variations from the characterized consensus, for example an insertion so large (see Fig. S1 and Fig. 7) that the fourth intron of the SF *RPL5* gene is only 17% conserved in relation to the consensus sequence of all three *Suberites* species. Many mechanisms and factors influence intron characteristics (e.g. see introduction in [53]). Up to date no genome of the *Suberites* genus has been sequenced, nor a ncRNAs library made, therefore it is very speculative to hypothesize on the origin of these insertions, i.e. if for example these insertions originating from mobile genetic elements and/or parts of the genome inserted during crossover.

The apparent lack of the majority of well defined human RPG-sited snoRNA orthologs in introns of sponge RPGs could be attributed either to non-conserved snoRNAs and their targets or snoRNA targets being well maintained and snoRNAs simply situated elsewhere in the genome. We checked 52 targets of the remaining 53 snoRNAs found in introns of RPGs in human and compared them with counterpart AQ rRNA sequences. Most (40) of these rRNA's target sequences are well conserved in sponge, three targets of three snoRNAs are unknown, two targets are not determined due to incomplete sponge 28S rRNA sequences, and seven are poorly conserved. Presence of the majority of target rRNA sequences lead us to seek out whether these snoRNA orthologs are nested somewhere else in the genome of AQ. We checked for orthologs of 19 C/D box snoRNAs with conserved target rRNA. Six conserved orthologs were found (see Fig. S2). This indicates that the total set of human RPG-sited snoRNA orthologs in sponge is probably higher than the set present in RPG introns which is in accordance with their dynamic nature.

From all these results we can conclude that sponge RPG introns share many structural characteristics with “higher” metazoans. These similarities are probably important because RPG introns function as carriers of snoRNAs. Intensive mobility of snoRNAs is probably the reason why sponges from different genera show discrepancy in snoRNA pools in orthologous RPG introns. Their mobile nature is also a plausible reason for almost equal RPG intron positions' conservation between sponges from different genus as between sponges and human, which indicates their more important role in diversification and enrichment of transcriptomes through mechanisms such as intron/exon gain/loss. Within the same genus large insertions in orthologous RPG introns were detected. Mechanisms of intron dynamics on this shorter evolutionary time scale is influenced by factors not correlated with mobility of snoRNAs.

## Supporting Information

Figure S1 **Percentage change from the consensus sequence of RPG introns from three species of the** ***Suberites*** **genus.** Introns (4L28 through 2L14 on the y axis) from *S. domuncula* (SD), *S. ficus* (SF) and *S. pagurorum* (SP), were aligned and the percent change from the resulting consensus sequence is shown per species. The average column shows the average percent change per intron for all three species. The last set of bars is the percent change from the consensus for the concatenated sequences of all introns from a species (CONCAT on the y axis).
(TIF)

Figure S2 **Human (HS) RPG-sited snoRNAs identified in sponge (AQ) introns of non-RP genes.** All essential snoRNA elements and methylation (Me) guide sites are designated.
(TIF)

Table S1 **Characteristics of 55 RPGs from seven model organisms.**
(DOC)

Table S2 **Sequences of primers used in this study.**
(DOC)

Table S3 **Characteristics of RPG introns in three basal metazoans.**
(DOC)

Table S4 **Comparison of 55 RPGs in ten organisms.**
(DOC)

Table S5 **Percentages of amino acid identity (above diagonal empty boxes) and overall similarity (below diagonal) extracted from GeneDoc.**
(DOC)

Table S6 **Percentages of intron positions shared among organisms.**
(DOC)

Table S7 **The sequences of the C/D and H/ACA box snoRNAs identified in RPG introns of sponges A. queenslandica (Aq), S. domuncula (Sd), S. ficus (Sf) and S. pagurorum (Sp).** Conserved snoRNA elements are boxed and predicted methylation guide sites are shaded. Experimentally verified snoRNAs are marked with asterisk (*). Lowercase letters indicate nucleotides that were not sequenced due to the snoRNA cloning strategy. Letters in brackets indicate nucleotides which do not belong to snoRNA, although they were predicted by snoSeeker.
(DOC)

Text S1 **126 introns sequences from three** ***Suberites*** **species:** ***S. domuncula*** **(SD),** ***S. ficus*** **(SF) and** ***S. pagurorum*** **(SP).**
(TXT)
