# Supplementary figures and images for: Structural and Functional Characterization of Ribosomal Protein Gene Introns in Sponges
Source: PLoS One. 2012 Aug 6;7(8):e42523. doi: 10.1371/journal.pone.0042523 (PMC3412847; doi:10.1371/journal.pone.0042523)

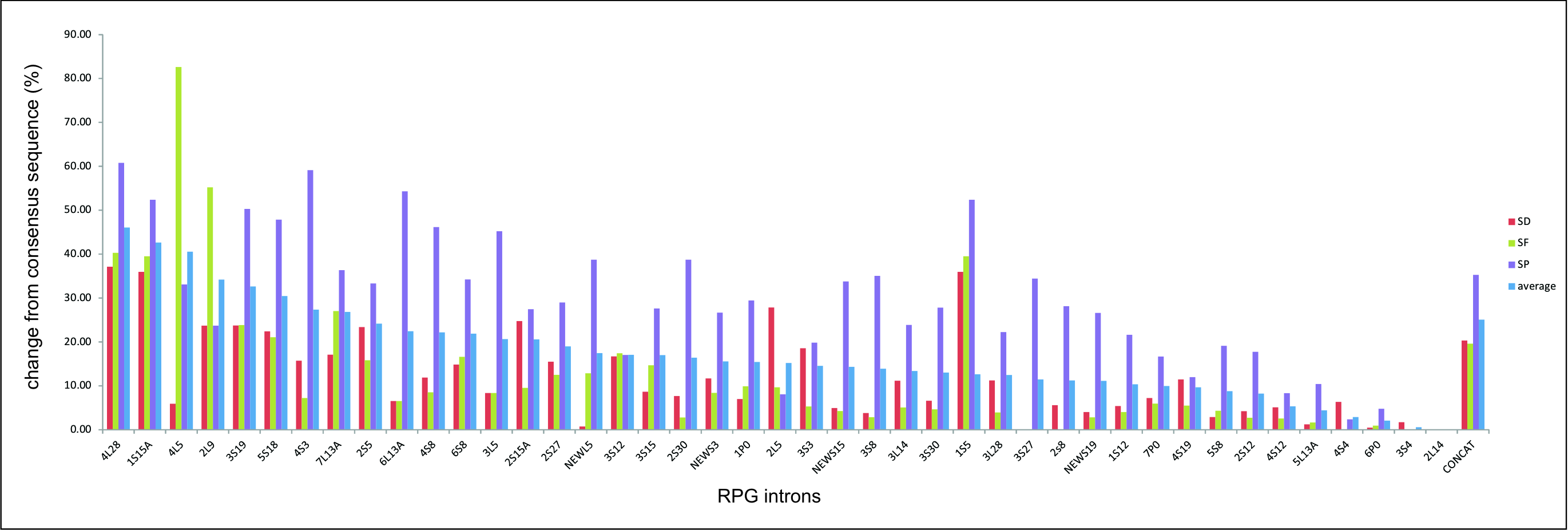

Supplement: Figure S1 — Percentage change from the consensus sequence of RPG introns from three species of the Suberites genus. Introns (4L28 through 2L14 on the y axis) from S. domuncula (SD), S. ficus (SF) and S. pagurorum (SP), were aligned and the percent change from the resulting consensus sequence is shown per species. The average column shows the average percent change per intron for all three species. The last set of bars is the percent change from the consensus for the concatenated sequences of all introns from a species (CONCAT on the y axis). (TIF) [file pone.0042523.s001.tif]

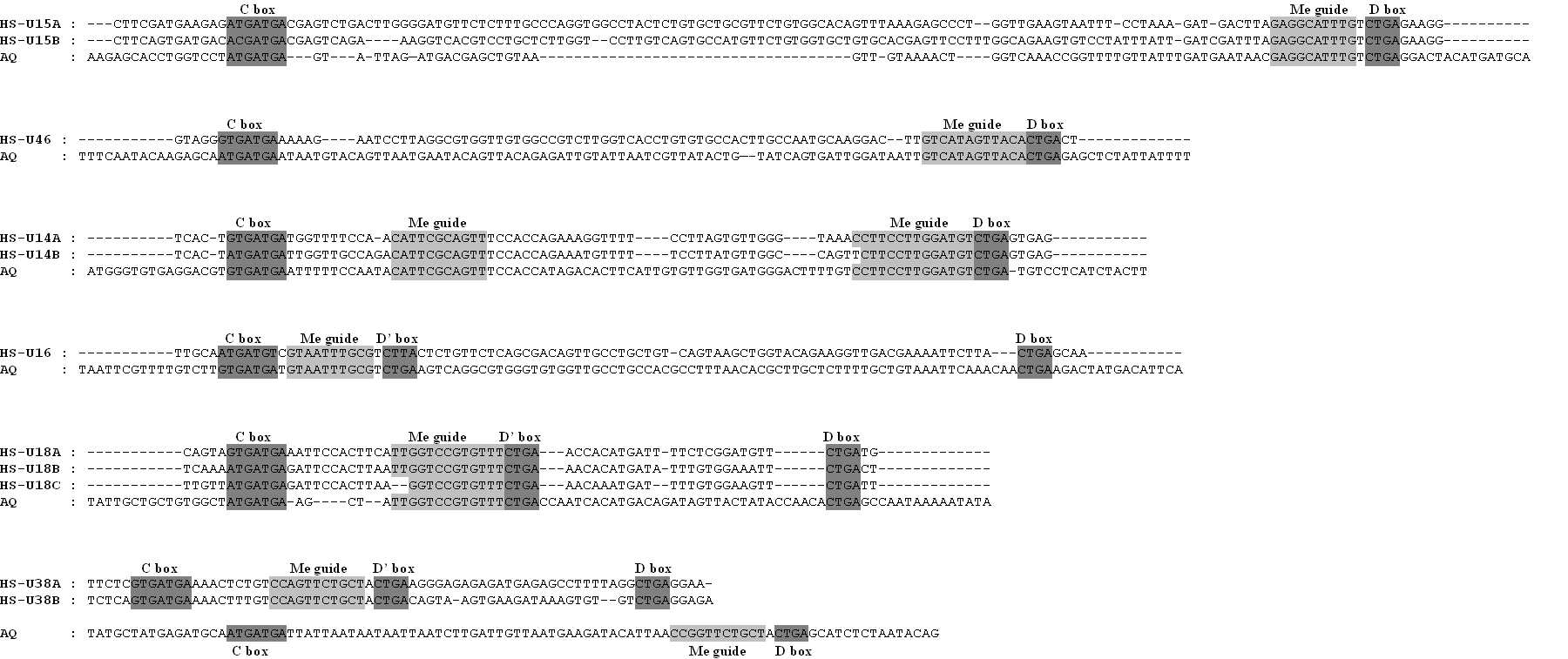

Supplement: Figure S2 — Human (HS) RPG-sited snoRNAs identified in sponge (AQ) introns of non-RP genes. All essential snoRNA elements and methylation (Me) guide sites are designated. (TIF) [file pone.0042523.s002.tif]
