# Supplementary material for: Structural and Functional Characterization of Ribosomal Protein Gene Introns in Sponges
Source: PLoS One. 2012 Aug 6;7(8):e42523. doi: 10.1371/journal.pone.0042523 (PMC3412847; doi:10.1371/journal.pone.0042523)
Supplement: Table S1 — Characteristics of 55 RPGs from seven model organisms. (DOC) [file pone.0042523.s003.doc]

**Supplemental Table S1. Characteristics of 55 RPGs from seven model organisms**

|  | **HS** | **HS** | **HS** | **DM** | **DM** | **DM** | **CE** | **CE** | **CE** | **SP** | **SP** | **SP** | **MB** | **MB** | **MB** | **SC** | **SC** | **SC** | **AT** | **AT** | **AT** |
| --- | --- | --- | --- | --- | --- | --- | --- | --- | --- | --- | --- | --- | --- | --- | --- | --- | --- | --- | --- | --- | --- |
| **RPG** | **Number of introns** | **CDS (bp)** | **Intron length (bp)** | **Number of introns** | **CDS (bp)** | **Intronlength (bp)** | **Number of introns** | **CDS (bp)** | **Intron length (bp)** | **Number of introns** | **CDS (bp)** | **Intron length (bp)** | **Number of introns** | **CDS (bp)** | **Intron length (bp)** | **Number of introns** | **CDS (bp)** | **Intron length (bp)** | **Number of introns** | **CDS (bp)** | **Intron length (bp)** |
| **SA** | 5 | 888 | 3827 | 1 | 813 | 192 | 3 | 831 | 166 | 6 | 1026 | 64527 | 1 | 837 | 86 | 1 | 759 | 359 | 4 | 897 | 798 |
| **S2** | 5 | 882 | 1646 | 0 | 804 | 0 | 2 | 819 | 96 | 4 | 846 | 1873 | 1 | 777 | 325 | 0 | 765 | 0 | 1 | 858 | 116 |
| **S3** | 5 | 732 | 4586 | 1 | 741 | 263 | 4 | 744 | 362 | 4 | 840 | 4080 | 2 | 759 | 1354 | 0 | 723 | 0 | 5 | 747 | 1091 |
| **S3A** | 5 | 795 | 4146 | 1 | 807 | 101 | 2 | 774 | 94 | 5 | 798 | 4758 | 2 | 780 | 397 | 0 | 768 | 0 | 6 | 789 | 710 |
| **S4** | 6 | 792 | 3733 | 4 | 786 | 1030 | 2 | 780 | 147 | 8 | 780 | 5925 | 3 | 795 | 590 | 1 | 786 | 269 | 4 | 789 | 607 |
| **S5** | 4 | 615 | 5998 | 3 | 687 | 2810 | 3 | 633 | 196 | 4 | 651 | 2508 | 3 | 642 | 356 | 0 | 678 | 0 | 3 | 624 | 536 |
| **S6** | 5 | 750 | 3153 | 2 | 747 | 748 | 2 | 741 | 227 | 4 | 738 | 3173 | 3 | 747 | 797 | 1 | 711 | 352 | 5 | 750 | 659 |
| **S7** | 5 | 585 | 4688 | 2 | 585 | 1435 | 3 | 585 | 225 | 4 | 591 | 3809 | 2 | 570 | 336 | 1 | 573 | 401 | 3 | 576 | 252 |
| **S9** | 3 | 585 | 5806 | 2 | 591 | 710 | 2 | 570 | 323 | 2 | 579 | 2350 | 3 | 591 | 445 | 1 | 594 | 501 | 2 | 597 | 614 |
| **S10** | 4 | 498 | 7218 | 0 | 492 | 0 | 2 | 450 | 100 | 3 | 495 | 2739 | 1 | 450 | 689 | 1 | 318 | 437 | 4 | 543 | 664 |
| **S11** | 4 | 477 | 2702 | 3 | 468 | 853 | 2 | 468 | 98 | 3 | 462 | 6196 | 3 | 480 | 500 | 1 | 471 | 511 | 5 | 480 | 697 |
| **S12** | 4 | 399 | 2375 | 2 | 420 | 709 | 2 | 423 | 396 | 3 | 435 | 3444 | 2 | 363 | 423 | 0 | 432 | 0 | 3 | 435 | 605 |
| **S13** | 5 | 456 | 2753 | 2 | 456 | 287 | 2 | 456 | 394 | 5 | 456 | 6404 | 2 | 462 | 1206 | 1 | 456 | 539 | 4 | 456 | 676 |
| **S15** | 3 | 438 | 1599 | 3 | 447 | 492 | 2 | 456 | 130 | 4 | 495 | 6044 | 3 | 435 | 565 | 0 | 429 | 0 | 3 | 459 | 398 |
| **S15A** | 3 | 393 | 5712 | 0 | 393 | 0 | 1 | 393 | 301 | 3 | 393 | 2290 | 3 | 390 | 332 | 1 | 393 | 483 | 2 | 393 | 260 |
| **S16** | 4 | 441 | 2182 | 4 | 447 | 1296 | 1 | 435 | 44 | 4 | 447 | 7727 | 3 | 432 | 942 | 1 | 432 | 432 | 1 | 441 | 131 |
| **S17** | 4 | 408 | 3220 | 2 | 396 | 428 | 2 | 393 | 209 | 4 | 513 | 6904 | 3 | 390 | 836 | 1 | 411 | 398 | 0 | 423 | 0 |
| **S18** | 5 | 459 | 3888 | 2 | 459 | 196 | 2 | 465 | 201 | 5 | 531 | 7314 | 2 | 465 | 819 | 1 | 441 | 435 | 3 | 459 | 715 |
| **S19** | 4 | 438 | 10163 | 1 | 471 | 102 | 2 | 441 | 244 | 2 | 444 | 1584 | 3 | 441 | 728 | 1 | 435 | 390 | 3 | 432 | 463 |
| **S20** | 3 | 360 | 934 | 3 | 363 | 612 | 3 | 354 | 185 | 2 | 360 | 1685 | 1 | 357 | 358 | 0 | 366 | 0 | 2 | 375 | 473 |
| **S23** | 3 | 432 | 1783 | 2 | 432 | 667 | 2 | 432 | 274 | 2 | 480 | 2379 | 2 | 432 | 333 | 1 | 438 | 320 | 3 | 429 | 688 |
| **S27** | 3 | 255 | 1042 | 2 | 255 | 831 | 1 | 252 | 315 | 3 | 255 | 7400 | 3 | 258 | 602 | 1 | 249 | 550 | 3 | 261 | 662 |
| **S27A** | 4 | 471 | 2282 | 1 | 471 | 189 | 2 | 492 | 335 | 3 | 471 | 3246 | 1 | 468 | 212 | 0 | 459 | 0 | 0 | 474 | 0 |
| **S28** | 2 | 210 | 335 | 0 | 195 | 0 | 1 | 198 | 55 | 1 | 198 | 2781 | 2 | 198 | 270 | 0 | 204 | 0 | 0 | 195 | 0 |
| **S30** | 3 | 402 | 731 | 1 | 399 | 87 | 2 | 393 | 172 | 3 | 414 | 3102 | 1 | 393 | 142 | 1 | 192 | 430 | 2 | 189 | 502 |
| **L3** | 9 | 1212 | 5446 | 5 | 1251 | 1670 | 3 | 1206 | 400 | 8 | 1215 | 6208 | 0 | 1260 | 0 | 0 | 1164 | 0 | 4 | 1170 | 655 |
| **L4** | 9 | 1284 | 4100 | 3 | 1224 | 668 | 3 | 1038 | 183 | 7 | 1158 | 4944 | 2 | 1095 | 297 | 0 | 1089 | 0 | 1 | 1224 | 473 |
| **L5** | 7 | 894 | 8857 | 3 | 900 | 751 | 3 | 882 | 145 | 6 | 891 | 9944 | 0 | 873 | 0 | 0 | 894 | 0 | 6 | 906 | 849 |
| **L6** | 5 | 867 | 2566 | 1 | 789 | 446 | 3 | 654 | 647 | 4 | 750 | 2235 | 2 | 705 | 448 | 1 | 531 | 415 | 3 | 702 | 565 |
| **L8** | 4 | 774 | 1552 | 1 | 771 | 193 | 2 | 783 | 282 | 4 | 774 | 2901 | 1 | 771 | 200 | 1 | 765 | 400 | 1 | 777 | 233 |
| **L9** | 5 | 579 | 3625 | 1 | 573 | 272 | 2 | 570 | 101 | 3 | 573 | 2187 | 2 | 690 | 808 | 0 | 576 | 0 | 2 | 585 | 653 |
| **L10** | 5 | 645 | 1690 | 3 | 657 | 927 | 2 | 645 | 94 | 4 | 651 | 3458 | 1 | 648 | 108 | 0 | 666 | 0 | 3 | 666 | 566 |
| **L11** | 5 | 537 | 4013 | 3 | 555 | 402 | 3 | 591 | 150 | 4 | 546 | 4806 | 0 | 591 | 0 | 0 | 525 | 0 | 4 | 549 | 604 |
| **L13A** | 7 | 612 | 3574 | 2 | 618 | 752 | 2 | 609 | 99 | 7 | 612 | 6743 | 3 | 612 | 566 | 1 | 600 | 290 | 3 | 621 | 751 |
| **L15** | 2 | 615 | 1027 | 1 | 615 | 173 | 2 | 615 | 419 | 3 | 615 | 2961 | 0 | 615 | 0 | 0 | 615 | 0 | 3 | 615 | 545 |
| **L17** | 5 | 555 | 2498 | 1 | 561 | 801 | 2 | 564 | 259 | 5 | 555 | 6601 | 2 | 528 | 255 | 1 | 555 | 306 | 6 | 528 | 891 |
| **L18** | 6 | 567 | 3205 | 2 | 567 | 361 | 4 | 567 | 291 | 4 | 492 | 2803 | 0 | 558 | 0 | 1 | 561 | 432 | 4 | 564 | 701 |
| **L18A** | 4 | 531 | 2776 | 1 | 534 | 422 | 1 | 543 | 48 | 4 | 531 | 3194 | 2 | 579 | 1302 | 1 | 519 | 477 | 3 | 537 | 379 |
| **L19** | 5 | 591 | 3713 | 2 | 612 | 310 | 3 | 597 | 286 | 4 | 597 | 3772 | 2 | 681 | 179 | 1 | 570 | 384 | 4 | 645 | 627 |
| **L21** | 4 | 483 | 2266 | 0 | 480 | 0 | 1 | 486 | 143 | 3 | 489 | 1512 | 1 | 486 | 538 | 1 | 483 | 388 | 1 | 495 | 563 |
| **L23A** | 4 | 471 | 3405 | 2 | 834 | 447 | 2 | 444 | 125 | 4 | 537 | 3067 | 4 | 429 | 957 | 1 | 429 | 414 | 2 | 465 | 391 |
| **L26** | 2 | 438 | 4309 | 0 | 450 | 0 | 2 | 429 | 326 | 2 | 444 | 569 | 2 | 423 | 419 | 1 | 384 | 447 | 0 | 441 | 0 |
| **L27** | 3 | 411 | 3759 | 0 | 408 | 0 | 2 | 411 | 95 | 3 | 411 | 2806 | 3 | 405 | 441 | 1 | 411 | 561 | 0 | 408 | 0 |
| **L27A** | 4 | 447 | 2546 | 2 | 450 | 461 | 2 | 438 | 446 | 3 | 486 | 3969 | 3 | 450 | 375 | 1 | 450 | 511 | 0 | 441 | 0 |
| **L29** | 2 | 480 | 1233 | 1 | 231 | 69 | 1 | 189 | 154 | 2 | 177 | 1386 | 2 | 180 | 278 | 0 | 180 | 0 | 1 | 186 | 137 |
| **L30** | 3 | 348 | 3219 | 1 | 336 | 405 | 2 | 342 | 240 | 3 | 345 | 4729 | 1 | 336 | 141 | 1 | 318 | 230 | 4 | 339 | 1005 |
| **L31** | 3 | 378 | 3302 | 1 | 375 | 70 | 1 | 369 | 325 | 3 | 375 | 2783 | 2 | 333 | 360 | 1 | 342 | 421 | 1 | 360 | 410 |
| **L32** | 2 | 408 | 3736 | 1 | 405 | 62 | 2 | 405 | 142 | 2 | 411 | 2210 | 2 | 402 | 288 | 0 | 393 | 0 | 1 | 402 | 359 |
| **L34** | 3 | 354 | 2899 | 1 | 489 | 62 | 1 | 333 | 127 | 4 | 342 | 2928 | 2 | 327 | 335 | 1 | 366 | 472 | 3 | 363 | 679 |
| **L36** | 2 | 318 | 796 | 1 | 348 | 64 | 1 | 315 | 50 | 2 | 342 | 1316 | 3 | 405 | 298 | 1 | 303 | 238 | 2 | 342 | 452 |
| **L36A** | 4 | 321 | 4384 | 2 | 315 | 523 | 2 | 318 | 265 | 4 | 315 | 6783 | 2 | 321 | 670 | 1 | 321 | 441 | 3 | 318 | 480 |
| **L37A** | 3 | 279 | 2251 | 2 | 279 | 322 | 0 | 276 | 0 | 3 | 426 | 4336 | 1 | 360 | 710 | 1 | 279 | 275 | 4 | 279 | 669 |
| **L38** | 3 | 213 | 5661 | 0 | 213 | 0 | 1 | 213 | 272 | 3 | 216 | 2851 | 3 | 210 | 656 | 0 | 237 | 0 | 2 | 210 | 798 |
| **P1** | 3 | 345 | 2214 | 1 | 339 | 145 | 1 | 336 | 49 | 3 | 336 | 2884 | 2 | 318 | 255 | 0 | 321 | 0 | 2 | 342 | 364 |
| **P2** | 3 | 348 | 2254 | 0 | 342 | 0 | 2 | 324 | 251 | 1 | 336 | 967 | 1 | 327 | 428 | 0 | 321 | 0 | 2 | 348 | 327 |
