# Supplementary material for: Structural and Functional Characterization of Ribosomal Protein Gene Introns in Sponges
Source: PLoS One. 2012 Aug 6;7(8):e42523. doi: 10.1371/journal.pone.0042523 (PMC3412847; doi:10.1371/journal.pone.0042523)
Supplement: Table S2 — Sequences of primers used in this study. (DOC) [file pone.0042523.s004.doc]

**Supplemental Table S2. Sequences of primers used in this study**

| **Oligonucleotides** | **Sequence (5’ → 3’)** |
| --- | --- |
| RPS3F | GTATGCAGAGAAGGTTGCAGCTAG |
| RPS3R | CACTTGGGATCGGCTGTTGTTGC |
| RPS4F | GCCTTGACTGCTGACGAGG |
| RPS4R | CAGGATCGGGGTAGCGGATGG |
| RPS5F | GTCTCTGTTGAGTTTGATGAGGTGG |
| RPS5R1 | GGTGAGACGTTCAACAATGGGAC |
| RPS5R2 | CTCCTTCACTGTGATGTAATCC |
| RPS8F1 | GCCGTATCCTCGACGTAGTCTAC |
| RPS8R1 | GTAGCCATCACATCGTCCACTCTG |
| RPS8F2 | GATAGTTGGCACAAGCGC |
| RPS8R2 | GAATGGAGTACAGTCCACC |
| RPS12F1 | CTTTCTTGTACGGACAAATC |
| RPS12R1 | GTTCGTTGCAGAGAGCCTCC |
| RPS12F2 | GGAGGCTCTCTGCAACGAAC |
| RPS12R2 | CTAAGCAGAAATGCTCTTG |
| RPS14F1 | GCTGCTATGTTGGCTGCCCAAGACG |
| RPS14R1 | GAGACGCCTTCCTCTTCTTCCTCCC |
| RPS14F2 | CAGCTAGGTCCACAGGTAGC |
| RPS14R2 | GTTCCTCCCGTAGCCCTAAG |
| RPS15F | CGTGGTGTTGACCTTGACC |
| RPS15R | GAATCTTGAAGAGTGTGTTGC |
| RPS15AF1 | GGTGCGTATGAACGTTTTGAACG |
| RPS15AR | CCAAGGATCTTCCCTCCAG |
| RPS15AF2 | GGAGAGTTTGAGATTGTTG |
| RPS18F1 | GACAAATTCCAGCATATTC |
| RPS18R1 | CTTCTTGGACACACCCACAG |
| RPS18F2 | CGAGAGAGTGATCACCATC |
| RPS18R2 | GAGAATACTTTCCATCAC |
| RPS19F | GACTGCCCGACTATGTTGAC |
| RPS19R | CTTTTTCTGCTTCTCTGACATCAC |
| RPS27F | CCAAAGATCTACTGCACCCC |
| RPS27R1 | CAATACGGCTTCCGTC |
| RPS27R2 | CCTGGACATTTTACATCC |
| RPL5F1 | GCCTTTTGTCAAGGTAATT |
| RPL5R1 | GCCTTGTACTTCTCTTCGTCC |
| RPL5F2 | GCCAAGATTGAAGGAGAC |
| RPL5R2 | GTCTCCTTCAATCTTGGC |
| RPL13AF1 | GAGGGGATCCCCCCACCTTACG |
| RPL13AR1 | CCAAGCTCAGCCAGTTGCTTG |
| RPL13AF2 | GATGTTGTGAAGACTCTTG |
| RPL13AR2 | GTACTTCCATCCAATTTC |
| RPP0F1 | GGGCAGGGAAGACAAAGCAGCG |
| RPP0R1 | GCCTCCTTGAAAGTAATGTCCG |
| RPP0F2 | CACTGAGGACGATCTAATC |
| RPP0R2 | GGTGTTCTTGCCCATAAGG |
| RPS4sno1 | GAATAACAAGGCTTAATT |
| RPS4sno2 | GAATAAACAAGGCTTAATA |
| RPS12sno1 | GTTACCAGTGATGATAC |
| RPS12sno2 | GTTACCAGTGATGATAT |
| RPS19sno | GACCATACCATGCTAATAC |
| RPL5sno | GTCCAATATTTGCTACTC |
| RPL13Asno1 | CTTGTGTGGTCATTGTCCA |
| RPL13Asno2 | CTTGTGTGGTCGTTGCACG |
| RPL28sno | CAATGATGAAATTAATTCGGTGAG |
| RPP0sno1 | GTGATGATAAATTGGATG |
| RPP0sno2 | GTGATGAAGAAGTGGGTG |
| modpolydT | GCGTAAGTGACTAGCGTGTTTTTTTTTTTTVN |
| uni | GCGTAAGTGACTAGCGTG |
