# Supplementary material for: Structural and Functional Characterization of Ribosomal Protein Gene Introns in Sponges
Source: PLoS One. 2012 Aug 6;7(8):e42523. doi: 10.1371/journal.pone.0042523 (PMC3412847; doi:10.1371/journal.pone.0042523)
Supplement: Table S3 — Characteristics of RPG introns in three basal metazoans. (DOC) [file pone.0042523.s005.doc]

**Supplemental Table S3. Characteristics of RPG** introns in three basal metazoans

|  | **AQ** | **AQ** | **AQ** | **AQ** | **AQ** | **TA** | **TA** | **TA** | **TA** | **TA** | **NV** | **NV** | **NV** | **NV** | **NV** |
| --- | --- | --- | --- | --- | --- | --- | --- | --- | --- | --- | --- | --- | --- | --- | --- |
| **RPG** | **CDS (bp)** | **GC content of CDS** | **Number of introns** | **Average intron length (bp)** | **GC content of introns** | **CDS (bp)** | **GC content of CDS** | **Number of introns** | **Average intron length (bp)** | **GC content of introns** | **CDS (bp)** | **GC content of CDS** | **Number of introns** | **Average intron length (bp)** | **GC content of introns** |
| **SA** | 939 | 0.465 | 8 | 50 | 0.301 | 861 | 0.452 | 6 | 124 | 0.339 | 933 | 0.558 | 4 | 603 | 0.344 |
| **S2** | 810 | 0.472 | 5 | 100 | 0.283 | 834 | 0.489 | 3 | 133 | 0.352 | 867 | 0.549 | 5 | 854 | 0.369 |
| **S3** | 711 | 0.483 | 4 | 404 | 0.356 | 687 | 0.42 | 5 | 401 | 0.369 | 723 | 0.529 | 4 | 882 | 0.357 |
| **S3A** | 777 | 0.434 | 5 | 114 | 0.304 | 777 | 0.399 | 5 | 279 | 0.363 | 792 | 0.497 | 5 | 402 | 0.341 |
| **S4** | 789 | 0.441 | 4 | 244 | 0.349 | 792 | 0.426 | 4 | 436 | 0.324 | 792 | 0.508 | 5 | 579 | 0.328 |
| **S5** | 621 | 0.471 | 6 | 168 | 0.338 | 612 | 0.429 | 4 | 168 | 0.346 | 630 | 0.53 | 5 | 711 | 0.375 |
| **S6** | 747 | 0.488 | 5 | 156 | 0.321 | 843 | 0.436 | 4 | 475 | 0.373 | 738 | 0.537 | 3 | 787 | 0.376 |
| **S7** | 585 | 0.414 | 6 | 167 | 0.335 | 576 | 0.4 | 8 | 215 | 0.34 | 576 | 0.476 | 8 | 333 | 0.372 |
| **S8** | 654 | 0.422 | 6 | 259 | 0.343 | 633 | 0.427 | 5 | 199 | 0.331 | 627 | 0.516 | 4 | 873 | 0.357 |
| **S9** | 582 | 0.47 | 3 | 77 | 0.363 | 585 | 0.397 | 2 | 520 | 0.354 | 573 | 0.528 | 2 | 3002 | 0.38 |
| **S10** | 483 | 0.435 | 6 | 139 | 0.341 | 486 | 0.439 | 5 | 357 | 0.366 | 501 | 0.53 | 4 | 979 | 0.365 |
| **S11** | 477 | 0.422 | 3 | 151 | 0.312 | 465 | 0.392 | 3 | 428 | 0.37 | 480 | 0.468 | 3 | 490 | 0.354 |
| **S12** | 408 | 0.449 | 5 | 278 | 0.317 | 438 | 0.439 | 5 | 289 | 0.339 | 426 | 0.52 | 5 | 1211 | 0.372 |
| **S13** | 456 | 0.426 | 5 | 51 | 0.308 | 456 | 0.435 | 5 | 216 | 0.376 | 456 | 0.468 | 6 | 355 | 0.35 |
| **S14** | 453 | 0.471 | 5 | - | - | 456 | 0.477 | 5 | 196 | 0.297 | 462 | 0.549 | 4 | 459 | 0.348 |
| **S15** | 441 | 0.463 | 3 | 588 | 0.369 | 438 | 0.418 | 2 | 408 | 0.362 | 450 | 0.51 | 2 | 760 | 0.338 |
| **S15A** | 393 | 0.392 | 3 | 229 | 0.359 | 393 | 0.387 | 4 | 292 | 0.375 | 393 | 0.467 | 4 | 1122 | 0.362 |
| **S16** | 438 | 0.446 | 4 | 152 | 0.292 | 426 | 0.442 | 4 | 157 | 0.31 | 426 | 0.534 | 5 | 716 | 0.36 |
| **S17** | 402 | 0.409 | 4 | 132 | 0.309 | 408 | 0.407 | 4 | 141 | 0.352 | 402 | 0.521 | 4 | 645 | 0.383 |
| **S18** | 459 | 0.425 | 5 | 183 | 0.332 | 462 | 0.429 | 5 | 243 | 0.322 | 462 | 0.492 | 4 | 895 | 0.381 |
| **S19** | 438 | 0.462 | 5 | 117 | 0.35 | 444 | 0.431 | 4 | 204 | 0.338 | 432 | 0.529 | 5 | 1038 | 0.355 |
| **S20** | 360 | 0.451 | 3 | 104 | 0.368 | 357 | 0.438 | 3 | 380 | 0.37 | 360 | 0.532 | 2 | 795 | 0.347 |
| **S21** | 252 | 0.426 | 4 | 94 | 0.299 | 255 | 0.421 | 4 | 187 | 0.298 | 255 | 0.472 | 5 | 428 | 0.334 |
| **S23** | 432 | 0.455 | 3 | 251 | 0.349 | 432 | 0.399 | 3 | 251 | 0.359 | 432 | 0.506 | 2 | 1265 | 0.367 |
| **S24** | 393 | 0.426 | 4 | 80 | 0.27 | 399 | 0.414 | 4 | 364 | 0.331 | 399 | 0.5 | 4 | 419 | 0.353 |
| **S25** | 363 | 0.467 | 2 | 53 | 0.232 | 330 | 0.401 | 2 | 532 | 0.348 | 351 | 0.537 | 2 | 870 | 0.342 |
| **S26** | 411 | 0.5 | 4 | 154 | 0.36 | 351 | 0.451 | 3 | 354 | 0.373 | 381 | 0.532 | 3 | 412 | 0.32 |
| **S27** | 255 | 0.421 | 3 | 788 | 0.367 | 255 | 0.437 | 3 | 576 | 0.38 | 255 | 0.496 | 3 | 1423 | 0.35 |
| **S27A** | 468 | 0.439 | 4 | 84 | 0.294 | 498 | 0.394 | 3 | 146 | 0.321 | 474 | 0.484 | 4 | 873 | 0.382 |
| **S28** | 195 | 0.453 | 2 | 47 | 0.32 | 198 | 0.456 | 2 | 500 | 0.345 | 198 | 0.497 | 2 | 528 | 0.367 |
| **S29** | 171 | 0.405 | 2 | 308 | 0.38 | 171 | 0.429 | 2 | 494 | 0.384 | 171 | 0.482 | 2 | 1692 | 0.357 |
| **S30** | 402 | 0.469 | 3 | 255 | 0.333 | 405 | 0.405 | 2 | 553 | 0.328 | 405 | 0.54 | 3 | 823 | 0.355 |
| **L3** | 1209 | 0.447 | 9 | 73 | 0.35 | 1200 | 0.414 | 8 | 293 | 0.388 | 1221 | 0.536 | 5 | 547 | 0.388 |
| **L4** | 1074 | 0.472 | 4 | 199 | 0.322 | 1050 | 0.444 | 4 | 126 | 0.296 | 1077 | 0.534 | 5 | 521 | 0.37 |
| **L5** | 906 | 0.436 | 6 | 186 | 0.338 | 894 | 0.43 | 7 | 247 | 0.357 | 891 | 0.493 | 7 | 677 | 0.382 |
| **L6** | 768 | 0.488 | 4 | 148 | 0.287 | 720 | 0.43 | 3 | 244 | 0.35 | 735 | 0.505 | 4 | 434 | 0.342 |
| **L7** | 753 | 0.435 | 5 | 203 | 0.349 | 738 | 0.386 | 4 | 309 | 0.338 | 738 | 0.479 | 4 | 834 | 0.378 |
| **L7A** | 834 | 0.437 | 6 | 82 | 0.342 | 798 | 0.411 | 7 | 178 | 0.336 | 792 | 0.493 | 5 | 530 | 0.355 |
| **L8** | 777 | 0.461 | 6 | 64 | 0.348 | 771 | 0.458 | 3 | 426 | 0.343 | 783 | 0.523 | 3 | 671 | 0.345 |
| **L9** | 567 | 0.431 | 4 | 288 | 0.279 | 570 | 0.397 | 4 | 375 | 0.347 | 570 | 0.469 | 4 | 441 | 0.346 |
| **L10** | 651 | 0.471 | 5 | 63 | 0.287 | 645 | 0.43 | 5 | 395 | 0.353 | 645 | 0.525 | 3 | 605 | 0.306 |
| **L10A** | 651 | 0.446 | 4 | 172 | 0.332 | 651 | 0.364 | 2 | 593 | 0.359 | - | - | - | - | - |
| **L11** | 546 | 0.434 | 3 | 99 | 0.294 | 510 | 0.432 | 2 | 163 | 0.298 | 501 | 0.502 | 3 | 493 | 0.352 |
| **L12** | 498 | 0.446 | 6 | 71 | 0.286 | 498 | 0.422 | 4 | 201 | 0.336 | 501 | 0.5 | 3 | 339 | 0.336 |
| **L13** | 648 | 0.45 | 3 | 98 | 0.268 | 636 | 0.444 | 3 | 312 | 0.317 | 636 | 0.524 | 3 | 783 | 0.343 |
| **L13A** | 609 | 0.422 | 7 | 224 | 0.335 | 609 | 0.396 | 5 | 344 | 0.346 | 606 | 0.498 | 7 | 457 | 0.347 |
| **L14** | 405 | 0.444 | 4 | 228 | 0.312 | 414 | 0.414 | 3 | 426 | 0.347 | 411 | 0.51 | 3 | 670 | 0.355 |
| **L15** | 615 | 0.459 | 3 | 141 | 0.262 | 615 | 0.458 | 2 | 624 | 0.36 | 615 | 0.539 | 2 | 573 | 0.35 |
| **L17** | 567 | 0.447 | 5 | 139 | 0.3 | 555 | 0.411 | 5 | 293 | 0.31 | 558 | 0.506 | 3 | 725 | 0.353 |
| **L18** | 564 | 0.442 | 4 | 92 | 0.276 | 564 | 0.431 | 6 | 151 | 0.324 | 567 | 0.514 | 6 | 496 | 0.389 |
| **L18A** | 531 | 0.402 | 4 | 581 | 0.355 | 528 | 0.417 | 4 | 371 | 0.343 | 531 | 0.492 | 4 | 533 | 0.352 |
| **L19** | 591 | 0.43 | 5 | 134 | 0.206 | 591 | 0.418 | 4 | 454 | 0.365 | 591 | 0.522 | 3 | 1049 | 0.369 |
| **L21** | 489 | 0.428 | 4 | 123 | 0.251 | 480 | 0.44 | 5 | 192 | 0.379 | 483 | 0.473 | 6 | 651 | 0.368 |
| **L22** | 402 | 0.430 | 3 | 156 | 0.328 | - | - | - | - | - | 372 | 0.442 | 3 | 610 | 0.373 |
| **L23** | 423 | 0.448 | 4 | 212 | 0.298 | - | - | - | - | - | - | - | - | - | - |
| **L23A** | 594 | 0.452 | 3 | 162 | 0.293 | 459 | 0.393 | 3 | 618 | 0.347 | 465 | 0.487 | 3 | 890 | 0.361 |
| **L24** | 474 | 0.467 | 3 | 271 | 0.317 | 453 | 0.416 | 4 | 254 | 0.329 | 465 | 0.539 | 4 | 1014 | 0.395 |
| **L26** | 441 | 0.427 | 2 | 63 | 0.205 | 438 | 0.428 | 1 | 1073 | 0.392 | 435 | 0.477 | 1 | 2253 | 0.328 |
| **L27** | 411 | 0.429 | 3 | 99 | 0.293 | 411 | 0.385 | 3 | 405 | 0.347 | 411 | 0.475 | 2 | 869 | 0.323 |
| **L27A** | 450 | 0.438 | 4 | 156 | 0.332 | 444 | 0.44 | 4 | 254 | 0.333 | 447 | 0.491 | 4 | 769 | 0.373 |
| **L28** | 426 | 0.423 | 3 | 335 | 0.303 | 423 | 0.44 | 3 | 467 | 0.339 | 414 | 0.513 | 4 | 697 | 0.348 |
| **L29** | 237 | 0.385 | 3 | 327 | 0.316 | 174 | 0.404 | 3 | 604 | 0.359 | 180 | 0.452 | 3 | 1455 | 0.367 |
| **L30** | 351 | 0.46 | 2 | 49 | 0.311 | 351 | 0.402 | 2 | 377 | 0.332 | 357 | 0.503 | 3 | 785 | 0.361 |
| **L31** | 372 | 0.423 | 4 | 446 | 0.348 | 369 | 0.418 | 4 | 208 | 0.338 | 372 | 0.493 | 4 | 637 | 0.345 |
| **L32** | 402 | 0.454 | 3 | 52 | 0.341 | 405 | 0.405 | 3 | 102 | 0.301 | 402 | 0.466 | 3 | 784 | 0.373 |
| **L34** | 348 | 0.446 | 4 | 97 | 0.328 | 351 | 0.443 | 5 | 202 | 0.355 | 345 | 0.506 | 5 | 729 | 0.371 |
| **L35** | 372 | 0.488 | 0 | - | - | 375 | 0.409 | 2 | 504 | 0.375 | 372 | 0.499 | 2 | 2098 | 0.381 |
| **L35A** | 333 | 0.412 | 4 | 188 | 0.287 | 348 | 0.409 | 2 | 424 | 0.331 | 342 | 0.525 | 3 | 722 | 0.364 |
| **L36** | 342 | 0.472 | 2 | 223 | 0.348 | 303 | 0.427 | 3 | 500 | 0.329 | 321 | 0.503 | 3 | 832 | 0.359 |
| **L36A** | 315 | 0.388 | 4 | 84 | 0.277 | 315 | 0.397 | 4 | 356 | 0.333 | 321 | 0.462 | 4 | 513 | 0.35 |
| **L37** | 348 | 0.496 | 2 | 256 | 0.291 | - | - | - | - | - | 303 | 0.537 | 2 | 901 | 0.356 |
| **L37A** | 279 | 0.428 | 3 | 74 | 0.27 | 279 | 0.42 | 3 | 501 | 0.338 | 279 | 0.493 | 3 | 329 | 0.353 |
| **L38** | 228 | 0.236 | 4 | 68 | 0.221 | 222 | 0.356 | 4 | 424 | 0.333 | 213 | 0.448 | 4 | 518 | 0.366 |
| **L39** | 156 | 0.410 | 2 | 44 | 0.292 | - | - | - | - | - | 156 | 0.484 | 2 | 805 | 0.328 |
| **L40** | 387 | 0.482 | 2 | 228 | 0.329 | - | - | - | - | - | 387 | 0.497 | 4 | 753 | 0.398 |
| **L41** | 78 | 0.427 | 2 | 48 | 0.272 | - | - | - | - | - | - | - | - | - | - |
| **P0** | 972 | 0.444 | 10 | 84 | 0.337 | 945 | 0.411 | 10 | 150 | 0.331 | 942 | 0.501 | 7 | 572 | 0.362 |
| **P1** | 345 | 0.497 | 3 | 55 | 0.244 | 333 | 0.47 | 2 | 287 | 0.361 | 333 | 0.561 | 2 | 2280 | 0.358 |
| **P2** | 354 | 0.481 | 3 | 48 | 0.326 | 339 | 0.443 | 2 | 214 | 0.333 | 342 | 0.54 | 2 | 950 | 0.364 |
| **Average** | **504** | **0.442** | **4.01** | **164** | **0.312** | **493** | **0.423** | **3.8** | **305** | **0.348** | **515** | **0.509** | **3.7** | **736** | **0.362** |

AQ, *A. queenslandica*; NV, *N. vectensis*; TA, *T. adhaerens*
