# Supplementary material for: Structural and Functional Characterization of Ribosomal Protein Gene Introns in Sponges
Source: PLoS One. 2012 Aug 6;7(8):e42523. doi: 10.1371/journal.pone.0042523 (PMC3412847; doi:10.1371/journal.pone.0042523)
Supplement: Table S4 — Comparison of 55 RPGs in ten organisms. (DOC) [file pone.0042523.s006.doc]

**Supplemental Table S4. Comparison of 55 RPGs in ten organisms**

| **Organism** | **Average CDS length (bp)** | **Number of introns** | **Average intron length (bp)** | **Ratio of species specific introns (%)** |
| --- | --- | --- | --- | --- |
| **HS** | 536 | 227 | 808 | 8.4 |
| **SP** | 539 | 203 | 1340 | 9.4 |
| **DM** | 539 | 88 | 282 | 15.9 |
| **CE** | 518 | 110 | 106 | 37.3 |
| **NV** | 523 | 182 | 837 | 4.4 |
| **TA** | 520 | 191 | 291 | 3.1 |
| **AQ** | 527 | 204 | 177 | 8.8 |
| **MB** | 524 | 106 | 235 | 39.6 |
| **SC** | 503 | 34 | 409 | 79.4 |
| **AT** | 525 | 146 | 188 | 48.6 |
