# Supplementary material for: Structural and Functional Characterization of Ribosomal Protein Gene Introns in Sponges
Source: PLoS One. 2012 Aug 6;7(8):e42523. doi: 10.1371/journal.pone.0042523 (PMC3412847; doi:10.1371/journal.pone.0042523)
Supplement: Table S5 — Percentages of amino acid identity (above diagonal empty boxes) and overall similarity (below diagonal) extracted from GeneDoc. (DOC) [file pone.0042523.s007.doc]

**Supplemental Table S5. Percentages of amino acid identity (above diagonal empty boxes) and overall similarity (below diagonal) extracted from GeneDoc**

|  | **HS** | **SP** | **DM** | **CE** | **NV** | **TA** | **AQ** | **SD** | **MB** | **SC** | **AT** |
| --- | --- | --- | --- | --- | --- | --- | --- | --- | --- | --- | --- |
| **HS** |  | 71 | 66 | 62 | 73 | 67 | 67 | 68 | 60 | 55 | 59 |
| **SP** | 81 |  | 63 | 60 | 70 | 65 | 64 | 65 | 58 | 53 | 57 |
| **DM** | 78 | 75 |  | 60 | 65 | 62 | 62 | 62 | 57 | 53 | 56 |
| **CE** | 75 | 73 | 74 |  | 61 | 59 | 59 | 59 | 56 | 53 | 54 |
| **NV** | 83 | 80 | 77 | 75 |  | 70 | 68 | 71 | 62 | 57 | 60 |
| **TA** | 79 | 77 | 74 | 74 | 82 |  | 66 | 67 | 60 | 55 | 58 |
| **AQ** | 79 | 77 | 75 | 73 | 81 | 79 |  | 74 | 59 | 54 | 58 |
| **SD** | 80 | 77 | 75 | 74 | 82 | 80 | 85 |  | 60 | 55 | 59 |
| **MB** | 74 | 71 | 71 | 71 | 75 | 74 | 73 | 74 |  | 55 | 55 |
| **SC** | 70 | 67 | 68 | 68 | 71 | 70 | 70 | 70 | 69 |  | 54 |
| **AT** | 73 | 70 | 70 | 70 | 74 | 73 | 72 | 73 | 70 | 69 |  |
