# Supplementary material for: Structural and Functional Characterization of Ribosomal Protein Gene Introns in Sponges
Source: PLoS One. 2012 Aug 6;7(8):e42523. doi: 10.1371/journal.pone.0042523 (PMC3412847; doi:10.1371/journal.pone.0042523)
Supplement: Table S6 — Percentages of intron positions shared among organisms. (DOC) [file pone.0042523.s008.doc]

**Supplemental Table S6. Percentages of intron positions shared among organisms**

|  | **HS** | **SP** | **DM** | **CE** | **NV** | **TA** | **AQ** | **MB** | **SC** | **AT** |
| --- | --- | --- | --- | --- | --- | --- | --- | --- | --- | --- |
| **HS** |  | 77.1 | 31.3 | 26 | 70.9 | 75.8 | 76.2 | 26 | 2.6 | 27.3 |
| **SP** | 86.2 |  | 31.5 | 25.6 | 67.5 | 71.9 | 73.4 | 25.6 | 3 | 26.1 |
| **DM** | 80.7 | 72.7 |  | 29.5 | 67 | 65.9 | 70.5 | 23.9 | 4.5 | 37.5 |
| **CE** | 53.6 | 47.3 | 23.6 |  | 42.7 | 45.5 | 44.5 | 11.8 | 0.9 | 20 |
| **NV** | 88.5 | 75.3 | 32.4 | 25.8 |  | 89.6 | 87.9 | 30.8 | 3.3 | 32.4 |
| **TA** | 90.1 | 76.4 | 30.4 | 26.2 | 85.3 |  | 89 | 28.3 | 3.1 | 31.4 |
| **AQ** | 84.8 | 73 | 30.4 | 24 | 78.4 | 83.3 |  | 27.9 | 2.9 | 29.9 |
| **MB** | 55.7 | 49.1 | 19.8 | 12.3 | 52.8 | 50.9 | 53.8 |  | 3.8 | 24.5 |
| **SC** | 17.6 | 17.6 | 11.8 | 2.9 | 17.6 | 17.6 | 17.6 | 11.8 |  | 17.6 |
| **AT** | 42.5 | 36.3 | 22.6 | 15.1 | 40.4 | 41.1 | 41.8 | 17.8 | 4.1 |  |
