# Supplementary material for: Structural and Functional Characterization of Ribosomal Protein Gene Introns in Sponges
Source: PLoS One. 2012 Aug 6;7(8):e42523. doi: 10.1371/journal.pone.0042523 (PMC3412847; doi:10.1371/journal.pone.0042523)
Supplement: Table S7 — The sequences of the C/D and H/ACA box snoRNAs identified in RPG introns of sponges A. queenslandica (Aq), S. domuncula (Sd), S. ficus (Sf) and S. pagurorum (Sp). Conserved snoRNA elements are boxed and predicted methylation guide sites are shaded. Experimentally verified snoRNAs are marked with asterisk (*). Lowercase letters indicate nucleotides that were not sequenced due to the snoRNA cloning strategy. Letters in brackets indicate nucleotides which do not belong to snoRNA, although they were predicted by snoSeeker. (DOC) [file pone.0042523.s009.doc]

**Supplemental Table S7. The sequences of the C/D and H/ACA box snoRNAs identified in RPG introns of sponges *A. queenslandica* (Aq), *S. domuncula* (Sd), *S. ficus* (Sf) and *S. pagurorum* (Sp). Conserved snoRNA elements are boxed and predicted methylation guide sites are shaded. Experimentally verified snoRNAs are marked with asterisk (*). Lowercase letters indicate nucleotides that were not sequenced due to the snoRNA cloning strategy. Letters in brackets indicate nucleotides which do not belong to snoRNA, although they were predicted by snoSeeker.**

| **snoRNA** | **Sequence** | **Location (ribosomal protein gene)** |
| --- | --- | --- |
| AqSNORD100 | TGATGTTACTGGCACAATGATGATAAATAGCTCCCTCTACTGATAATTATTGTGAGGATAAAAAGCTTAACCGGAATCACTATATACACTGATGTGTCATTTTAATT | RPS12 |
| SdSNORD100a | TTATATGGAGTTACCAGTGATGATACATAGAGCTCCCTCTACTGAATATTAGTGTAGAAATGCTCATTAACCGGAATCACTATACTGTCTGAGGTGTGCTCCTATTG | RPS12 |
| SdSNORD100b* | ccatatggaGTTACCAGTGATGATATATAGAGCTCCCTCTACTGAATAATAGTGTGGAAATGCTCATTAACCGGAATCACTATTAAATCTGAGGTG(TGCTCCATATA) | RPS12 |
| SfSNORD100a | TTATATGGAGTTACCAGTGATGATACATAGAGCTCCCTCTACTGAATATTAGTGTAGAAATGCTCATTAACCGGAATCACTATACTGTCTGAGGTGTGCTCCTATTG | RPS12 |
| SfSNORD100b* | ccatatggaGTTACCAGTGATGATACATAGAGCTCCCTCTACTGAATAATAGTGTGGAAATGCTCATTAACCGGAATCACTATTAAATCTGAGGTGT(GCTCCATATA) | RPS12 |
| SpSNORD100a | TTATATGGAGTTACCAGTGATGATACATAGAGCTCCCTCTACTGAATATTAGTGTAGAAATGCTCATTAACCGGAATCACTATACTGTCTGAGGTGTGCTCCTCTTG | RPS12 |
| SpSNORD100b* | aattatggaGTTACCAGTGATGATACATAGAGCTCCCTCTACTGAATAATAGTGTAGAAATGCTCATTAACCGGAATCACTATAAAATCTGAGGTG(TGCTCCATATA) | RPS12 |
| AqSNORD24 | TGTTGTATAATGGCCGGTGATGTAAACTTATACTTGCTACTCTTGATCTTGTTAAGTGATGATTGTATACCACCAAGATCTCTGAGGCCATTCTATCATA | RPL5 |
| SdSNORD24* | gtatgttaaagagctaATGATGTCCAATATTTGCTACTCTTGATACCAGGAGCACACGTTCCTGGGAATGATGAGTACTTCACCATATCTGAAGC(TCTTGGAATTTG) | RPL5 |
| SfSNORD24* | gtatgttaaagagctaATGATGTCCAATATTTGCTACTCTTGATACCAGGAGCACACGTTCCTGGGAATGATGAGTACTTCACCATATCTGAAGC(TCTTGGAATTTG) | RPL5 |
| SpSNORD24* | acaaagttaggagctgATGATGTTCAATATTTGCTACTCTTGATACCAGGAGCACAAGTTCTTGGGAATGATGAGTACTTCACCATAATCTGAAGC(TCTTGGAATTTG) | RPL5 |
| AqSNORD83 | TCACTCGCTAGTGCAAATGATGAATCAATGGTTATATGTCACAGCTGATGTTGTTTATGTTGATATTATTCCATTGCTGCCTTCCTTCTGATGCACTTTAATAAAT | RPP0 |
| SdSNORD83a* | gtatgtgtggttgtttGTGATGATAAATTGGATGTTATTACTAGACAGAGTTCAAACATTGACGATCACATCCAATACTGCCTTCCTTCTGAACCAA(CCCAATGAAA) | RPP0 |
| SdSNORD83b | CCATTTTGATTTGTTTGTGATGAAGAAGTGGGTGTTTTTACTAGACAGAGTTTAAACAGTGACGATCACATCCAATACTGCCTTCCTTCTGAAACACCTATAAGTAC | RPP0 |
| SfSNORD83a* | gtagttaaggttgtttGTGATGATAAATTGGATGTTATTACTAGACAGAGTTCAAACATTGACGATCACATCCAATACTGCCTTCCTTCTGAAACAA(CCCAATGAAA) | RPP0 |
| SfSNORD83b | CCATTTTGATTTGTTTGTGATGAAGAAGTGGGTGTTTTTACTAGACAGAGTTTAAACAGTGACGATCACATCCAATACTGCCTTCCTTCTGAAACACCTATAAGTAC | RPP0 |
| SpSNORD83a* | gtgtgtccggttgtttGTGATGATACTCTGGATGTTATTACTAGACAGAGTTCAAACAGTGACGATTACATCCTATACTGCCTTCCTTCTGAAACAA(CCCAATGAAA) | RPP0 |
| SpSNORD83b | CCATTTTGATTTGTTTGTGATGAAGAAGTGGGTGTTTTTACTAGACAGAGTTTAAACAGTGACGATCACATCCAATACTGCCTTCCTTCTGAAACACCTATAAGTAC | RPP0 |
| AqACA1 | ACGAGACAGAGTCACATGATCAGCTACTTTCTTCGTGTTGTTTGTGTGCTAAGTACTCGTTATATCAACCAGTTATTATTGATATCCACACTCCTGGATTGATTGTTAGTTCCATGAAGTGTCACCTATACGCTGACATTC | RPL13A |
| SdACA1a | TACGAGGGGTCTAGAGTCACTTGTGTGGTCATTGTCCAAACTATTCATGTGCTACCCCCTCGTGATATCAATTGCTCTGTTATTCGCTGAATAGACCTATCTTCAATGTGGTCTACTTGGTCACCAACTCAGCATACAAAA | RPL13A |
| SdACA1b* | tacgaggggtctagagtcaCTTGTGTGGTCGTTGCACGAACTATTCATGTGCTACCCCCTCGTGATATCAATTGCTCTGTTATTCGCTGAATAGACCTATCAATGTGGTCTACTTGGTCACCAACTCAGCATACACAA | RPL13A |
| SfACA1a | TACGAGGGGTCTAGAGTCACTTGTGTGGTCATTGTCTAAACTATTCATGTGCTACCCCCTCGTGATATCAATTGCTCTGTTATTCGCTGAATAGGCCTATCTTCAATGTGGTCTACTTGGTCACCAACTCAGCATACAAAA | RPL13A |
| SfACA1b* | tacgaggggtctagagtcaCTTGTGTGGTCGTTGCACAAACTATTCATGTGCTACCCCCTCGTGATATCAATTGCTCTGTTATTCACTGAATAGACCTATCAATGTGGTCTACTTGGTCACCAA  CTCAGCATACACAA | RPL13A |
| SpACA1a | TACGAGGGGTCTAGAGTCACTTGTGTGGTCGCTGCTCAAACTATTCATGTGCTACCCCCTCGTGATATCAATTGCTCTGTTATTCGCTGAATAGACCTATCTTCAATGTGGTCTACTTGGTCACCAACTTTGCATACAAAA | RPL13A |
| SpACA1b* | tacgaggggtctagagtcaCTTGTGTGGTCGTCGCCCCAAACTATTTCATGTGCTACCCCCTCGTGATATCAATTGCTCTGTTATTCGCTGAATAGACCTATCTTCAATGTGGTCTACTTGGTCACCAACTCAGCATACACCA | RPL13A |
| AqCD1 | GTAAATATAATGAATGATGATGATGGATGTAATTTGTATGATTTCTGTATTTCACCCAATAAATGAGTCTCAAAACTAGCTTTTGTGTATACACGTACATGTCATGTAAAGAATATGGCCTAACTTAGCTTATTCTGTTGCTGCTGATTTCTGTTGATGAAA | RPS3 |
| AqCD2 | TATGTACATGTATGCTATGATGACTGGAATAATCCCAGCTCGCTTTGAGCTCTGGCTAAGAGTGTTGAAGAGAGACAGTTCCTTCCATTCTGAGCACCACATGTATTG | RPS4 |
| AqCD3 | TTTTATATAGAGGATAATGATGATCACGTGATGCCCCCATATGATCACAAATTAAATGATTTTAAAAGTCACGTCATATATTATATAATGGCTGAATCCTCTCAACCACA | RPS5 |
| AqCD4 | TTACACAGCAACGAAAATGATGCAATCAGTATAGTTTTGTGCCTGTTTTATGCTTCTGTTTACACAGAAACGAATGAAAACCTGATAGAATCTTGATATG | RPS8 |
| AqCD5 | GTCATTACACATAATTATGAGGAGCAGTAATGAGGAGCAATGGGCTGACTAAAGCCAGATTTACAATATGACGCTTGTCCTGACGTTTTGTTGACACAGAAGTTGAATCTGGATCAACTCCAGCCTGAAGTATGCGTCAAAAG | RPS14 |
| AqCD6 | GTTACTATACCTATTAATGATTAAATAGGAGGGTAGGAGAATTTTGTTGCTAATGTTGACTAATGTATTGCTGAAAGTATTGGCGAAAG | RPS15 |
| AqCD7 | GTGAGTGGCAAAGTGTTGAAATTCAATATGTCACTTCTTTTTTATAAATGTGGGACATTCATACAGTAATACAGTTGTGTAGTAAAAGGGGAGCTGAGTGCCGCTTATTACT | RPS15A |
| AqCD8 | TTTATTAATGATTAAAATGATGAAGTTATTTATGTGTAATTAGAATTATATTGGTAGTATGTGAATAGTTATGTGTATTGAATGAGGAATGGTGAATGTGACTCTTGATATTAGAAAAATCATTAGGCTTCTGATATTAATGATCAAAT | RPS18 |
| AqCD9 | TTCCTATTGTTGGCTAATGATGTTTACCATGCTAATGCTGATGGGTGTGGTTCTGTGGTTGCCATAGTAACTGCAAACCGGCTCCTGTGTGAGAGTAAATAATATGACCTGAAGCCACTAATGCTGC | RPS19 |
| AqCD10 | AAGAAAAGCAAGTACTAGGATGAGTAATGGAAGCCCTAAAAATAAACAGTAAAACTCAAAAACAGGCAAAACTGAAAATTATAAATGTGA | RPS27 |
| AqCD11 | AAATACCATTTGGCCGATGATTAATAATTGCATGTATTACCTGACTGGTCATGTGACTGTCACATGACCTGATGCTCATCCTTACCT | RPP0 |
| AqCD12 | GTAATATAATGATGATTACTGTTATTGTATTGTAGTGCCTCAGTTTGGTGAATTTCTAAGTTAGTTTAAATCAATCAATTTAGAATTTGTATCTGAAGTTAGGAATATTTT | RPL14 |
| AqCD13 | TTATAATTTTTTTCTGATGCATATACATGTTTTATGTAGTGTAGCAAGTTTGAGCTGATTCTGATCAAAATTAATT | RPL28 |
| SdCD12a* | atagagatatttgacaGTGATGAATAACAAGGCTTAATTTCTGAGCTCATGTGAGTGAGCTGTGATCACCTTGTAATACTCTGAGTCA(CTTGTTAACCA) | RPS4 |
| SdCD12b | GTGGTGTTGGTGTGTTGTGATGAATAAACAAGGCTTAATATCTGAGTTCATTCTGTGAACTGTGATCACTTTGTTACTCTGAATGCATCAGGATATC | RPS4 |
| SfCD12a* | atagagatatttgacaGTGATGAATAACAAGGCTTAATTTCTGAGCTCATGTGAGTGAGCTGTGATCACCTTGTAATACTCTGAGTCA(CTTGTTAACCA) | RPS4 |
| SfCD12b | GTGGTGTTGGTGTGTTGTGATGAATAAACAAGGCTTAATATTTGAGTTCATTCTGTGAGCTGTGATCACTTTGTTACTCTGAATGCATCAGGATATC | RPS4 |
| SpCD12a* | atagagatatttgacaGTGATGAATAACAAGGCTTAATTTCTGAGCTCATGTGAGTGAGCTGTGATCACCTTGTAATACTCTGAGTCA(CTTGTTAACCA) | RPS4 |
| SpCD12b | GTGGTGTTGGTGTGTTGTGATGAATAAACAAGGCTTAATATTTGAGTTCATTCTGTGAGCTGTGATCACTTTGTTACTCTGAATGCATCAGGATATC | RPS4 |
| SdCd13* | ggtcagtgttaaactaATGATGACCATACCATGCTAATACAGAGCTTTTGCTTTGATGACAGCATAACGGTTTCTGAAGTTTAA(CAATTCTC) | RPS19 |
| SfCd13* | ggtcagtgttatactaATGATGACCATACCATGCTAATACAGAGCTTTTGCTTTGATGACAGCATAACGGTTTCTGAAGTTTAA(CAATTCTC) | RPS19 |
| SpCd13* | ggtcagtgttaaactaATGATGACCATACCATGCTAATACAGAGCTTTTGCTTTGATGACAGCATAACGGTTTCTGAAGTTTA(CAATTACTC) | RPS19 |
| SdCd14* | tttgttgttctgccCAATGATGAAATTAATTCGGTGAGACACGTGGTAGAGCCTGGGTTGTTTCTTACCTTTTATCTTTCTTTTTCTCCTCCTTTCTACATTCCCCGCCCCACTTTTCTGTTCTAGAACTTTCTTTTCCCCCAATTTTTCATTTTCTTCCCTCCTTCCCATGGCTCTGTCGATGATAGTTAAGATTATGAGAGCTGAGGGCA(GCTGTAGTAA) | RPL28 |
| SfCd14 | TTTGTTGTTCTGCCCAATGATGAAATTAATTCGGTGAGACACGTGGTAGAGCCTGGGTTGTTTCTTACCTTTTATCTTTCTTTTTCTCCTCCTTTCTAAATTCCCCGCCCCACTTTTCTGTTCTAGAACTTTCTTTTCCCCCAATTTTTCATTTTCCTCCCTCCTTCCCATGGCTCTGTCGATGATAGTTAAGATTATGAGAGCTGAGGGCAGCTGTAGTAA | RPL28 |
| SpCd14 | TTTGTTGTTCTGCCCAATGATGAAAATAATTCGGTGAGACACGTGGTAGAGCCTGGGTTGTTTCTTACCTTTTTTCTTTCTTTTTCTCCTCCTTTCTACCTTCCCCACCCCACTTTTCTGTTCTAGAACTTTCTTTCCTCCCGTTTTTCATTTTTCTTCCTCCTTCCCATGGCTCTGTCGATGATAGTTAAGATTATGAGAGCTGAGGGCAGCTGTAGTAA | RPL28 |
| AqACA2 | AAGTTATTATTTATTTATTAATGGGTGTGGTTGAGAGGATTCAGCCATTATATAATATATGACGTGACTTTTAAAATCATTTAATTTGTGATCATATGGGGGCATCACGTGATCATCATTATCCTCTATATAAAATAATTATAATATTAATGACAAAC | RPS5 |
